# Supplementary material for: Dynamic contrast enhancement and flexible odor codes
Source: Nat Commun. 2018 Aug 3;9:3062. doi: 10.1038/s41467-018-05533-6 (PMC6076288; doi:10.1038/s41467-018-05533-6)
Supplement: Supplementary file 1 — Supplementary Information [file 41467_2018_5533_MOESM1_ESM.pdf]

SUPPLEMENTARY INFORMATION:

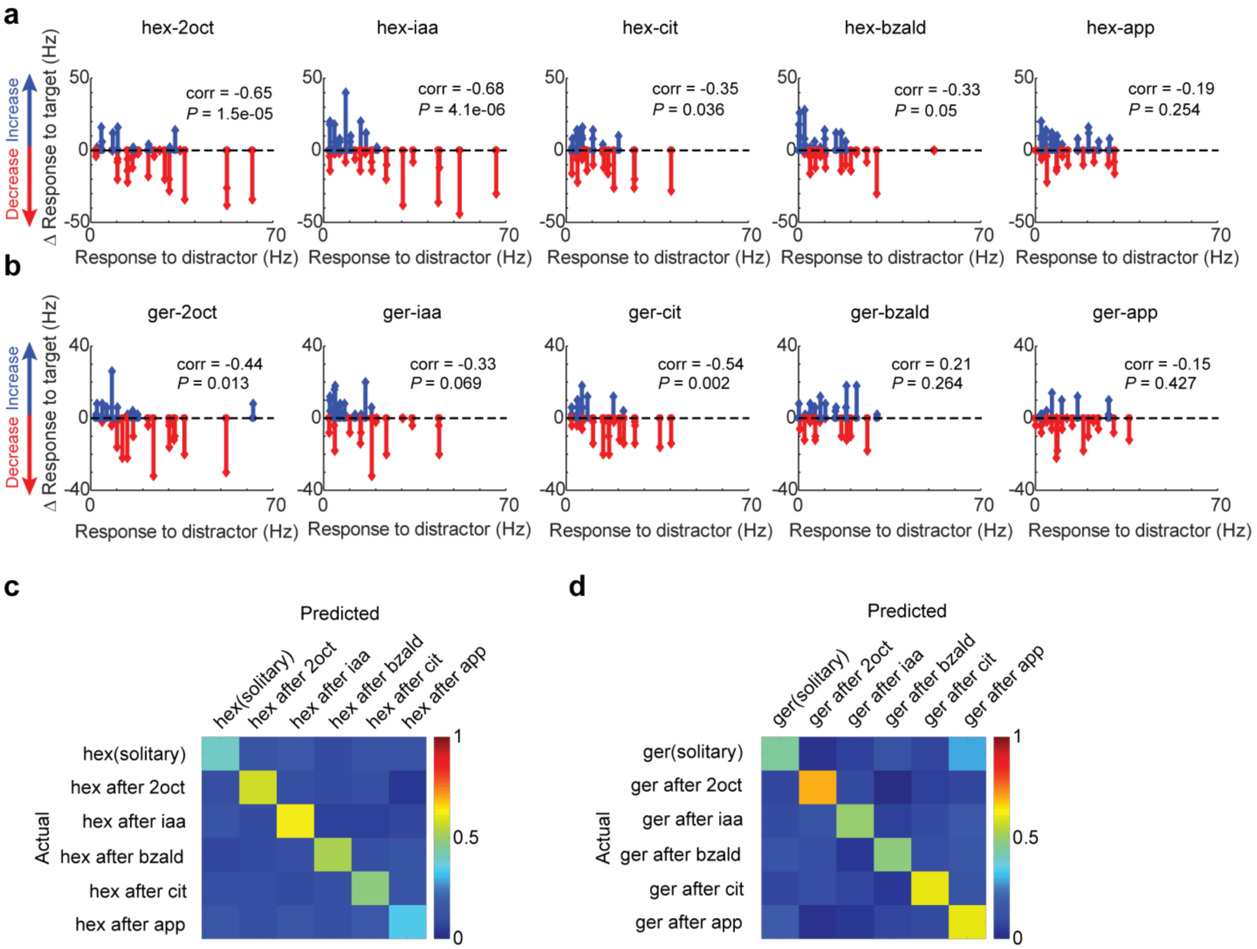

Supplementary Figure 1

(a) Change in PN responses to the target odorant (y axis) vs. response to distractor (x axis) is plotted for five distractor-target pairs (the target odorant is hex in all cases). The maximum spike rate in a 50 ms time bin during the first 1 s of odor presentation is shown for all PNs that were excited by hex (*n* = 36 PNs for solitary hex; see Methods for PN response categorization). Zero represents identical response to both solitary and sequential presentation of the target odorant. Red lines indicate that the response to target after distractor is less than the response to target alone (i.e. negative values). Blue lines indicate increase in the response for target after distractor when compared to target alone (i.e. positive values). A very small uniform random noise has been added to jitter the points with same x-values and reduce overlap between colored lines. Correlations between the change in target odor response and response to distractor odor (corr values) and their significance levels are shown on each panel.

(b) Similar plots as **panel a** but plotted when the target odor was ger (*n* = 30 excitatory PNs for solitary ger).

(c) The separability of the PN responses (six categories: hex(solitary) and five sequential conditions of hex) is quantified and shown as a confusion matrix. Rows correspond to the actual stimulus identity and the columns indicate the predicted stimulus identity. A nearest centroid

method with leave-one-out cross-validation in 85-D space was used for generating these classification results (see Methods). Note that the confusion matrix is mostly diagonal indicating that the PN responses evoked by the same stimulus presented with different histories are distinct.

(d) Similar plot as in **panel c** but the confusion matrix analyzing the response separability of the solitary and sequential geraniol presentations is shown.

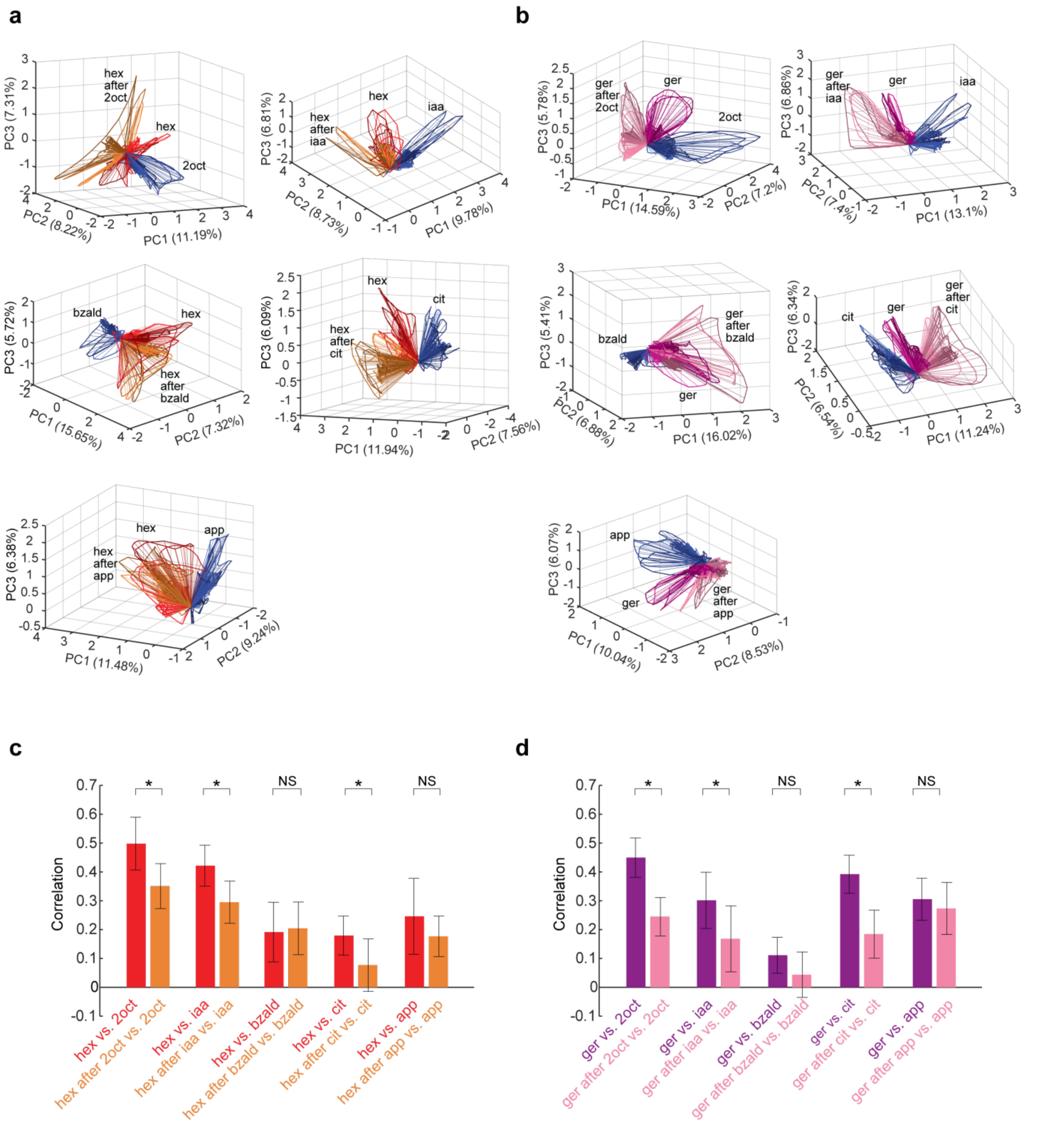

Supplementary Figure 2

(a) Similar trajectory plots as shown in **Fig. 2a,b** but trial-to-trial variations are included. Population PN responses evoked by the distractor odorant, hex, and the sequential presentation of hex are shown for three sets of trials: mean of trials 1-3, mean of trials 4-6, and mean of trials 7-10. The darker colored traces correspond to the earlier set of trials.

(b) Similar plots as in **panel a** but shown for five sequential presentations of geraniol.

(c, d) Similar plots as in **Fig. 2c,d**, but the correlations are now computed using the mean odor-evoked response during the last 1 s of stimulus presentation window.

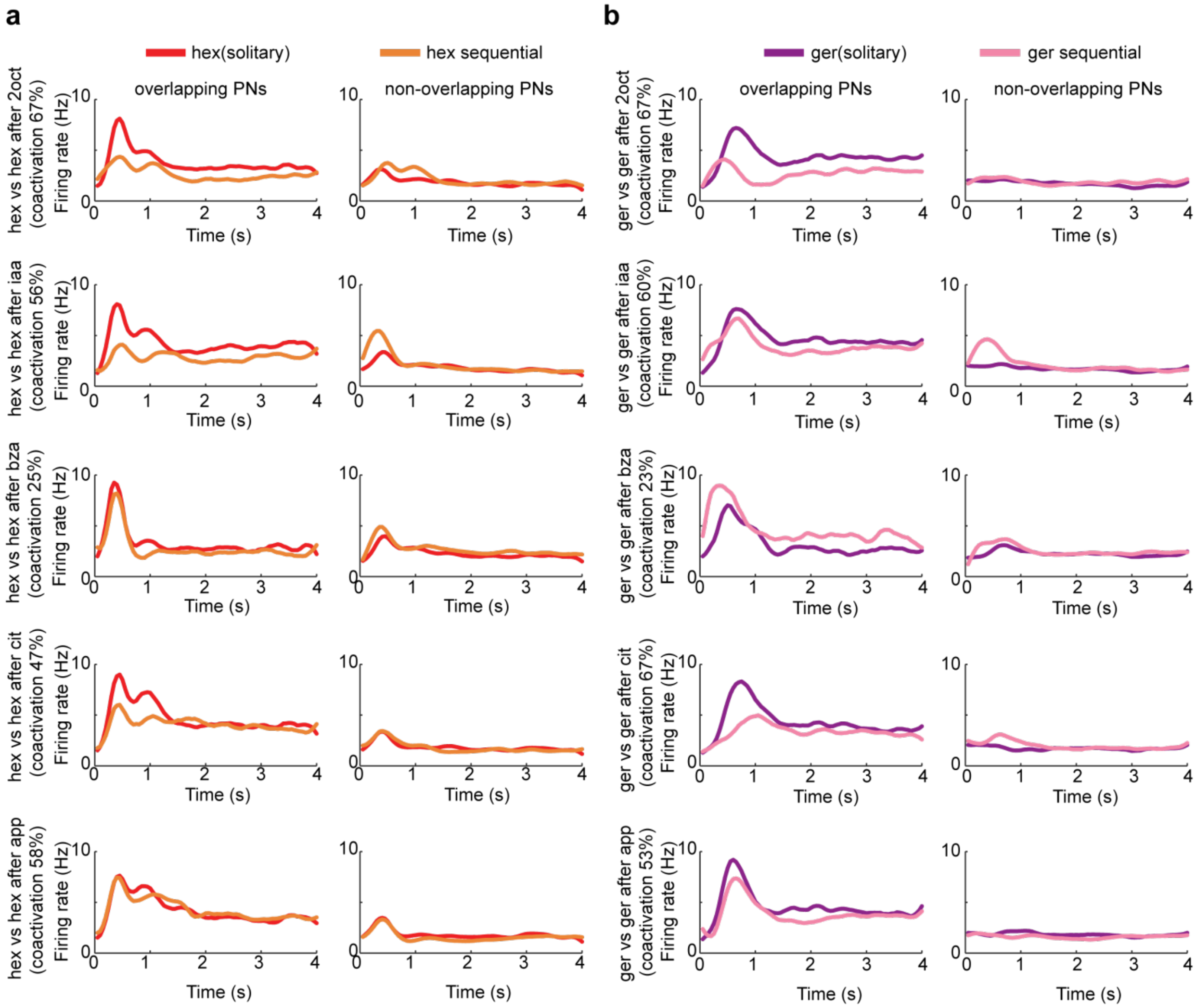

Supplementary Figure 3

(a) Comparison between the mean firing rates averaged across two distinct sets of PNs is shown for the solitary (red trace) and sequential presentations (orange traces) of hex. Overlapping PNs correspond to the set of PNs that were responsive to both the target (hex) and distractor odorant. 'Non-overlapping PNs' correspond to the remaining set of PNs that were not 'overlapping PNs'. Left panel shows the firing rate of overlapping PNs averaged across trials (*n* = 10). Right panel shows the firing rate of non-overlapping PNs averaged across ten trials. The percentage of overlapping PNs (i.e. co-activation) for each distractor odorant is shown.

(b) Similar plots as in **panel a** but comparing the mean firing rates of overlapping and non-overlapping PNs during solitary and sequential geraniol presentations.

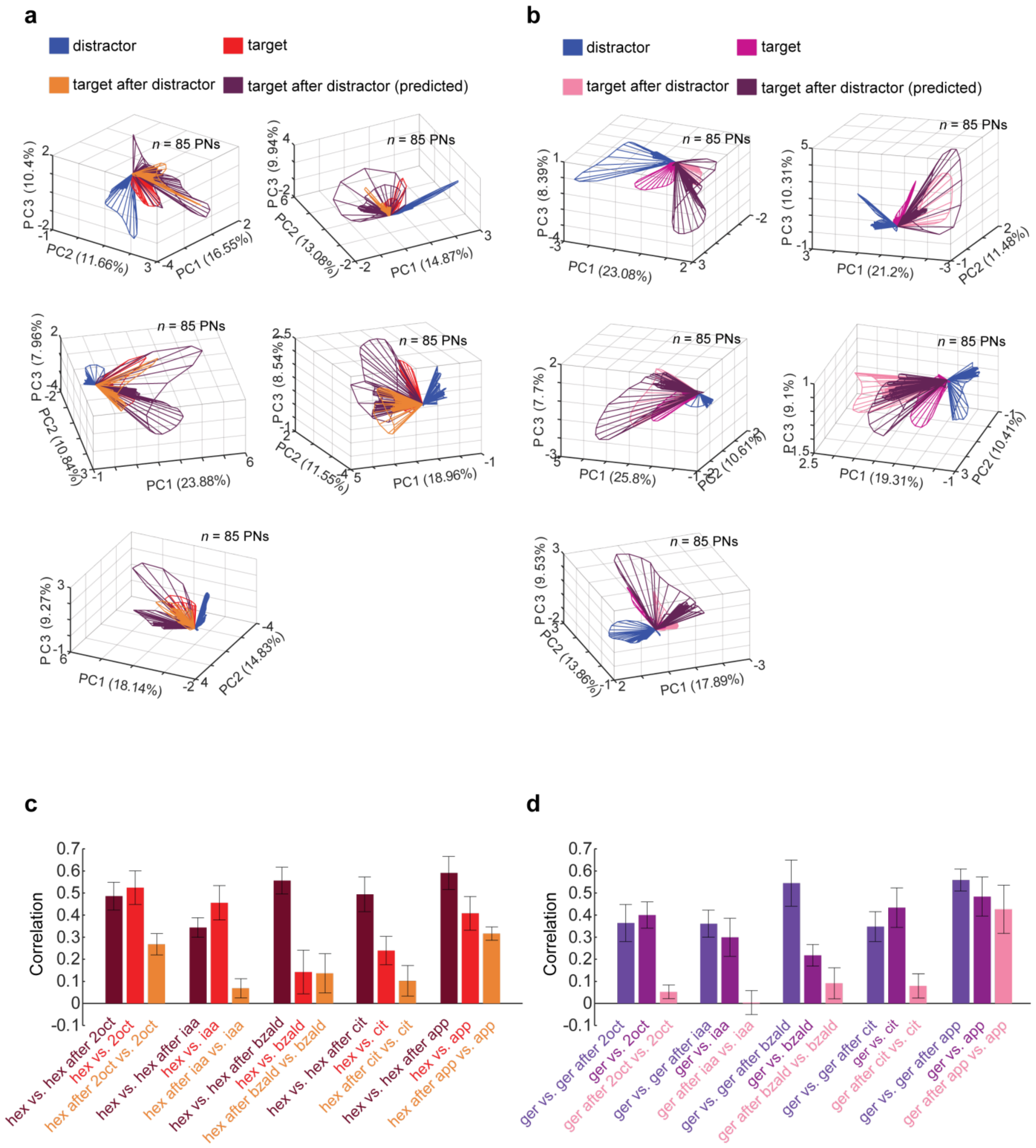

Supplementary Figure 4

(a, b) Similar plots as in **Fig. 2a, b.** Predicted response trajectory is shown in each panel along with response trajectories for distractor, target, and target after distractor. The predictions were made for each target after distractor by taking a sum of target vector and (target vector - distractor vector) at each time bin.

(c, d) Similar plots as in **Fig. 2c, d**, but correlations between target alone and target after distractor are also shown for comparison.

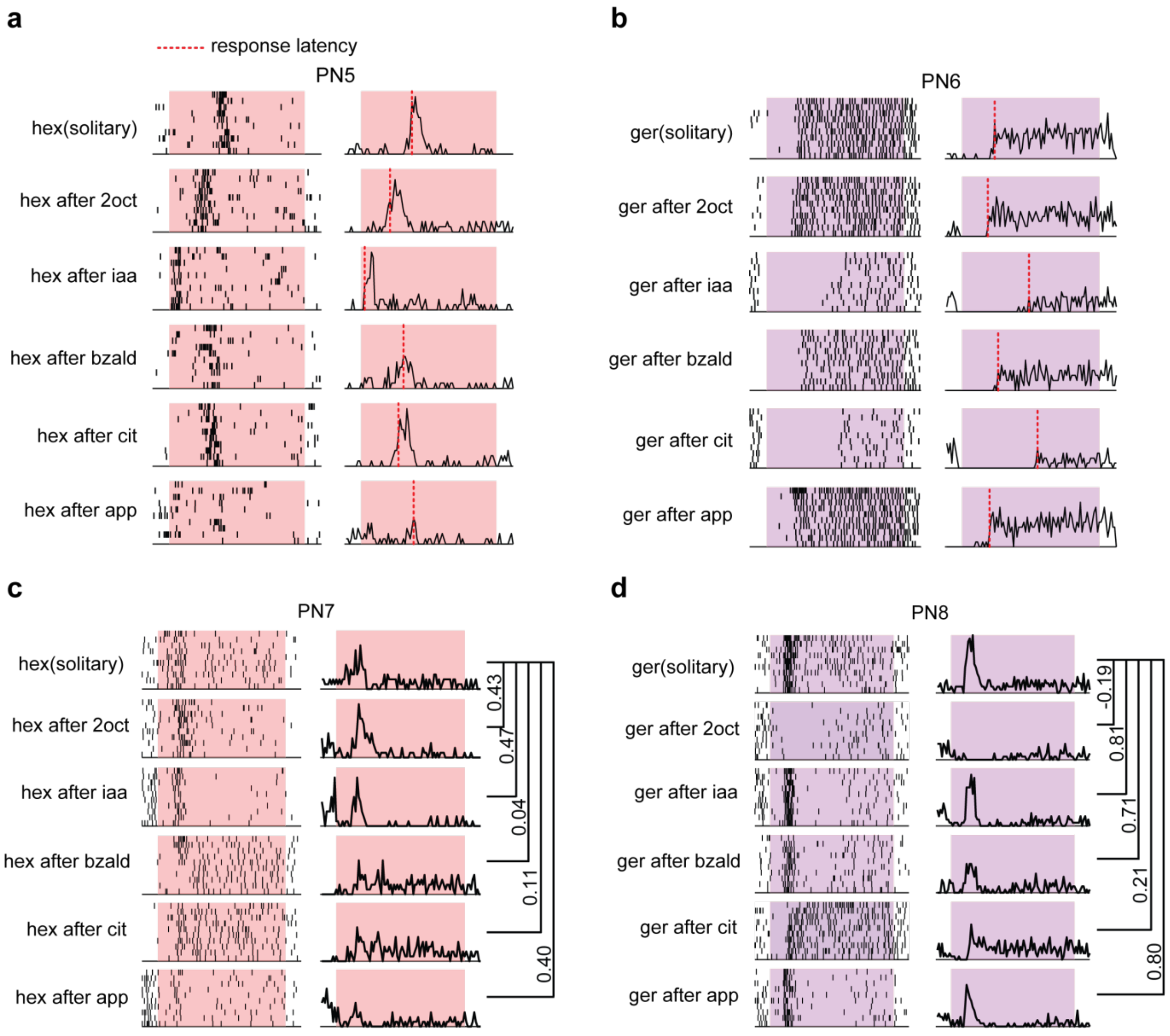

Supplementary Figure 5

(a) Left panel, raster plots showing spiking response of a PN to solitary and sequential introductions of hexanol. Same conventions as **Fig. 1c**. Right panel, mean spike counts in 50 ms time bins across trials are plotted as a function of time. The first time bin when the firing rate exceeds a fixed threshold was defined as its response latency and is identified using a dotted vertical line in each panel.

(b) Similar plots as in **panel a** but showing the response of a different PN to solitary and sequential introductions of geraniol. The evolution of firing rates over time and the response latency for each geraniol presentation are shown.

(c) Left panel, raster plot showing a representative PNs spiking responses to different hexanol introductions. Right panel, mean spike counts plotted as a function of time. Correlations between firing rate patterns observed during different sequential hexanol introductions with solitary hexanol presentations are shown.

(d) Similar plot as in **panel c** showing the spiking response variations for different geraniol introductions.

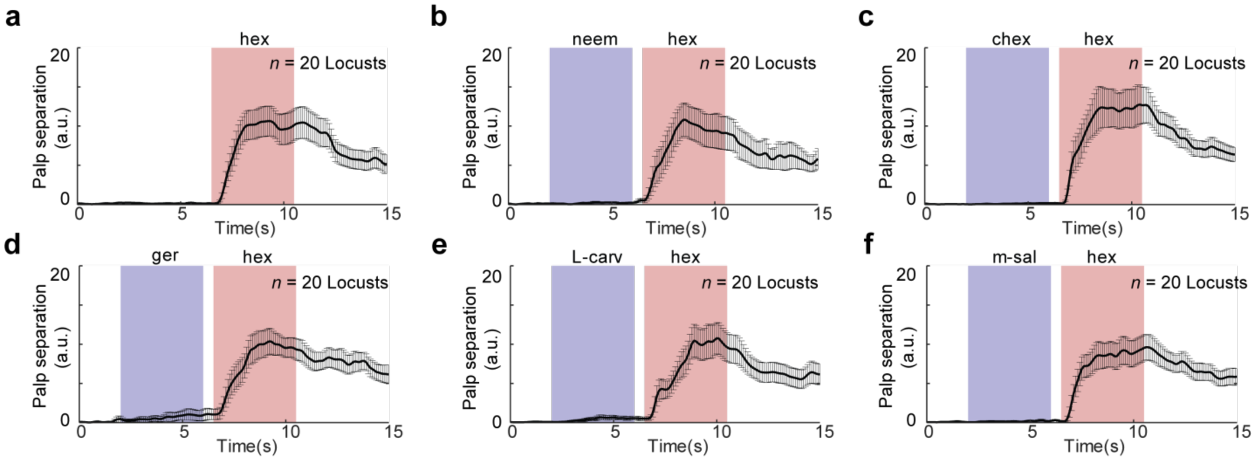

Supplementary Figure 6

(a-f) Palp-opening responses (POR) to additional distractor-target odor sequences are shown. The distance between the palps was tracked and plotted as a function of time. Error bar represents standard error across locusts (n = 20).

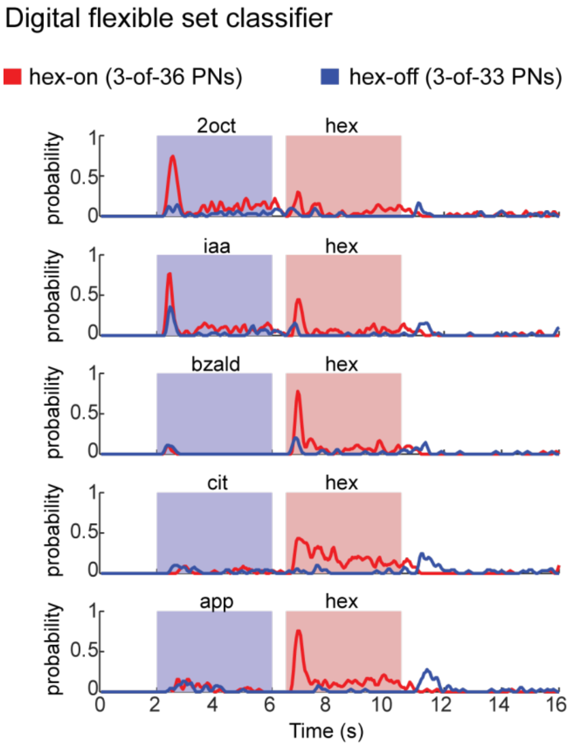

Supplementary Figure 7

Classification results for the digital version of the flexible set decoder are shown. For any 50 ms time bin, the threshold for ON classification was set to be 3–of–36 PNs must be responding to the stimulus presentation (i.e. firing rate > 6.5 s.d. of pre-stimulus activity). The threshold for OFF classification was also set to be 3–of–33 PNs. The 36 hex-ON PNs and 33 hex-OFF PNs were determined based on solitary hexanol presentations alone. Note that these classification results are very similar to the analog version that we presented in **Fig. 7b**.

a

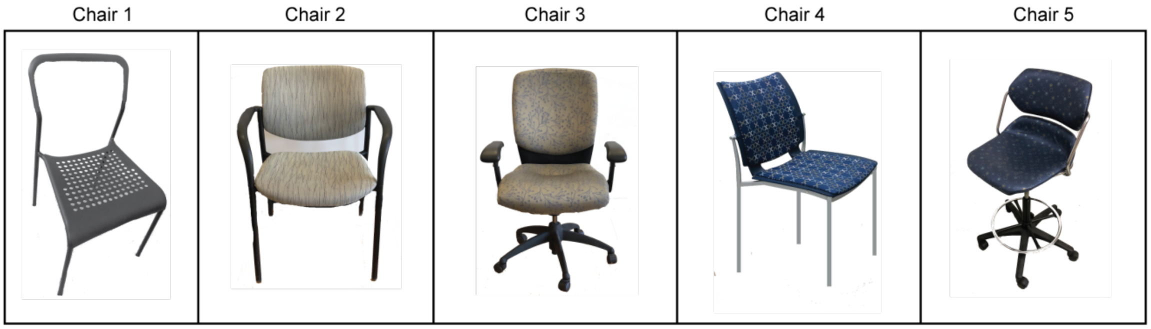

b

Feature / Object	4 Legs	Seat	Back support	Arm rest
Chair 1	✓	✓	X	X
Chair 2	✓	✓	✓	✓
Chair 3	X	✓	✓	✓
Chair 4	✓	✓	✓	X
Chair 5	X	✓	✓	X

✓ → 1

X → 0

c

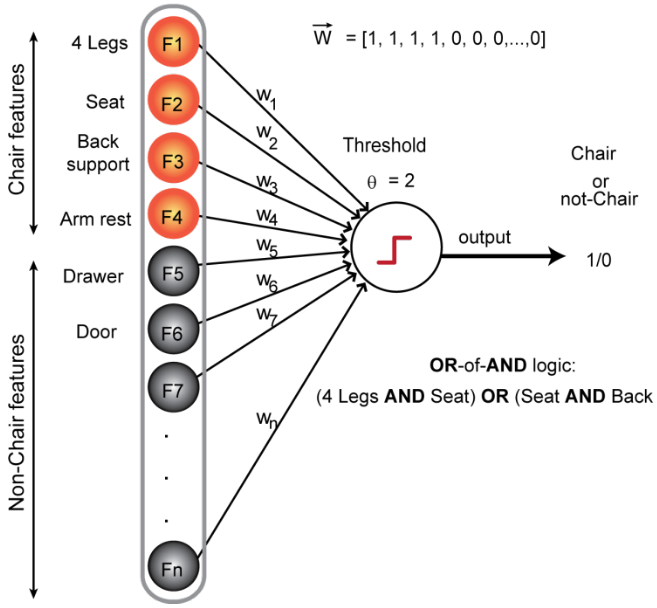

OR-of-**AND** logic:

(4 Legs **AND** Seat) **OR** (Seat **AND** Back support) **OR** … (4 Legs **AND** Seat **AND** Back support **AND** Arm rest)

d

Flexible object recognition	Flexible odor decoding
Chair	Target stimulus (hex or ger)
Different chairs	Different presentations of the same target stimulus
Feature present	PN responding
Feature absent	PN not responding
Chair recognition	Stimulus identification

Supplementary Figure 8

(a, b) A schematic illustration of the flexible set decoder is shown. Here the problem is cast as one of object recognition (i.e. chair recognition). Images of different chairs and their features are tabulated. Photo courtesy of Raman Lab.

(c) A schematic of an OR-of-ANDs or disjunction-of-conjunction classifier is shown. Input features are binary 'feature present' or 'feature absent'. The weight vectors are constant and set based on an ideal object (Chair 2 in **panel a**): the weight vector component is a '*1*' if the feature is present in the ideal chair, and '0' if it not present. The only free parameter in the OR-of-ANDs classifier is the threshold of the output node (θ). If the value of the threshold is set below the total number of features present in the ideal object (for example $\theta = 2$), then the presence of any two of the four features will allow recognition of the object (i.e. flexible decoding). This can be written as a set of logical OR-of-ANDs operation.

(d) The list of analogies between this object/chair recognition illustration and flexible odor decoding proposed in this manuscript are listed in a table.

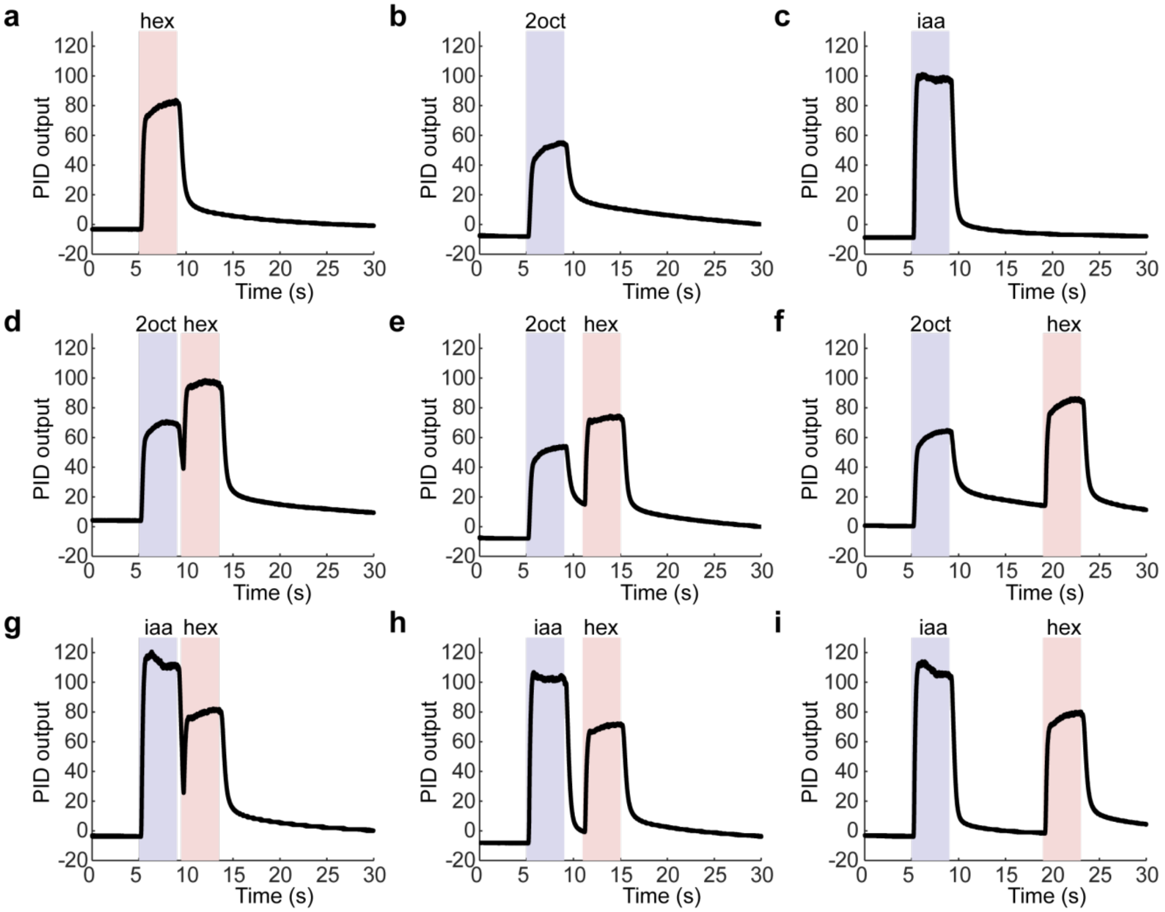

Supplementary Figure 9

(a, b, c) Photoionization detector (PID) measurements are shown. The color bars indicate when a 4 s odor puff was presented. The three traces shown correspond to hex, 2oct and iaa presentations, respectively. Mean across five trials is plotted in each panel.

(d, e, f) Similar PID traces are shown for 2oct-hex (distractor-target) odor sequences with three different lags between the two stimuli: 0.5 s, 2 s, and 10 s.

(g, h, i) Similar plots as **panels d, e, f,** but for a different distractor-target (iaa-hex) odor sequence.

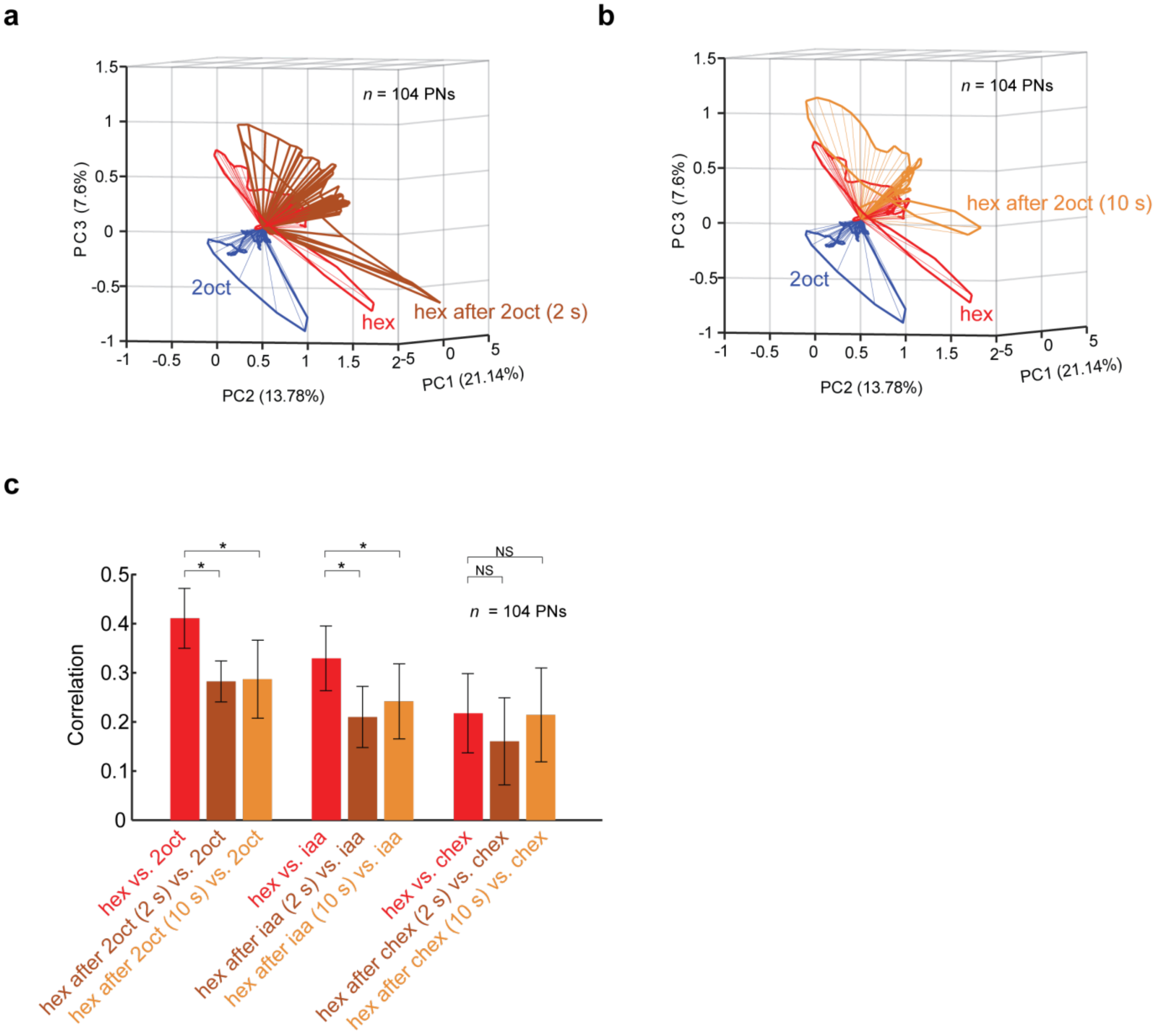

Supplementary Figure 10

(a, b) Response trajectories generated by two additional sequential presentations of hex are plotted: 2oct – 2 s – hex and 2oct – 10 s – hex. Similar method as in **Fig. 2a** was followed to analyze this dataset.

(c) The mean of correlation values between the ensemble PN responses (*n* = 104 PNs) evoked by hexanol and the three distractor cues (2oct, iaa, and chex) are shown as bar plots. Error bars indicate ± s.d across ten trials. The mean odor-evoked responses during the initial 1 s after stimulus onset were used for computing these correlations. The odor-pairs that were compared are identified along the x-axis. Asterisks indicate a significant decrease in the correlation (**P* < 0.025 (Bonferroni corrected for two comparisons), t-tests, *n* = 10 trials).

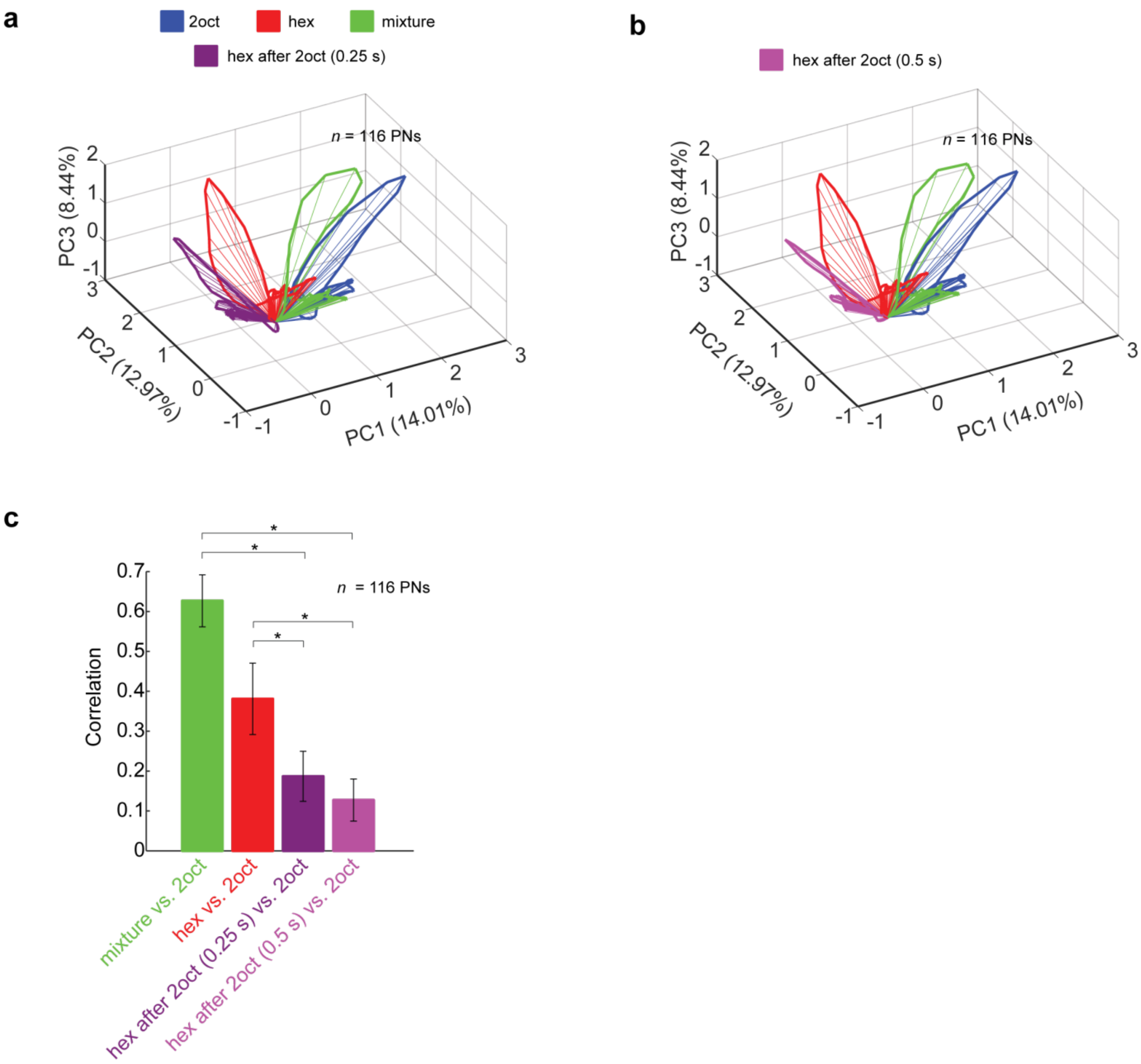

Supplementary Figure 11

(a, b) Similar plots as in **Fig. 2a** but showing ensemble response trajectories for the following stimuli: hex (red), 2oct (blue), a binary mixture of hex and 2oct (hex-2oct; green), 2oct – 0.25 s – hex (purple; top panel) and 2oct – 0.5 s – hex (purple; bottom panel). These data were re-analyzed from our previous study[1].

(c) Correlation between binary mixture response, hex response, hex after 2oct (0.25 s) response, and hex after 2oct (0.5 s) response with 2oct-evoked neural activity were calculated and plotted as a bar graph (mean ± s.d; n = 10 trials). Mean ensemble activity during the initial 1 s after stimulus onset was used to compute these correlations. Asterisks indicate a significant decrease in the correlation (*$P < 0.0125$ (Bonferroni corrected for four comparisons), t-tests, n = 10 trials).

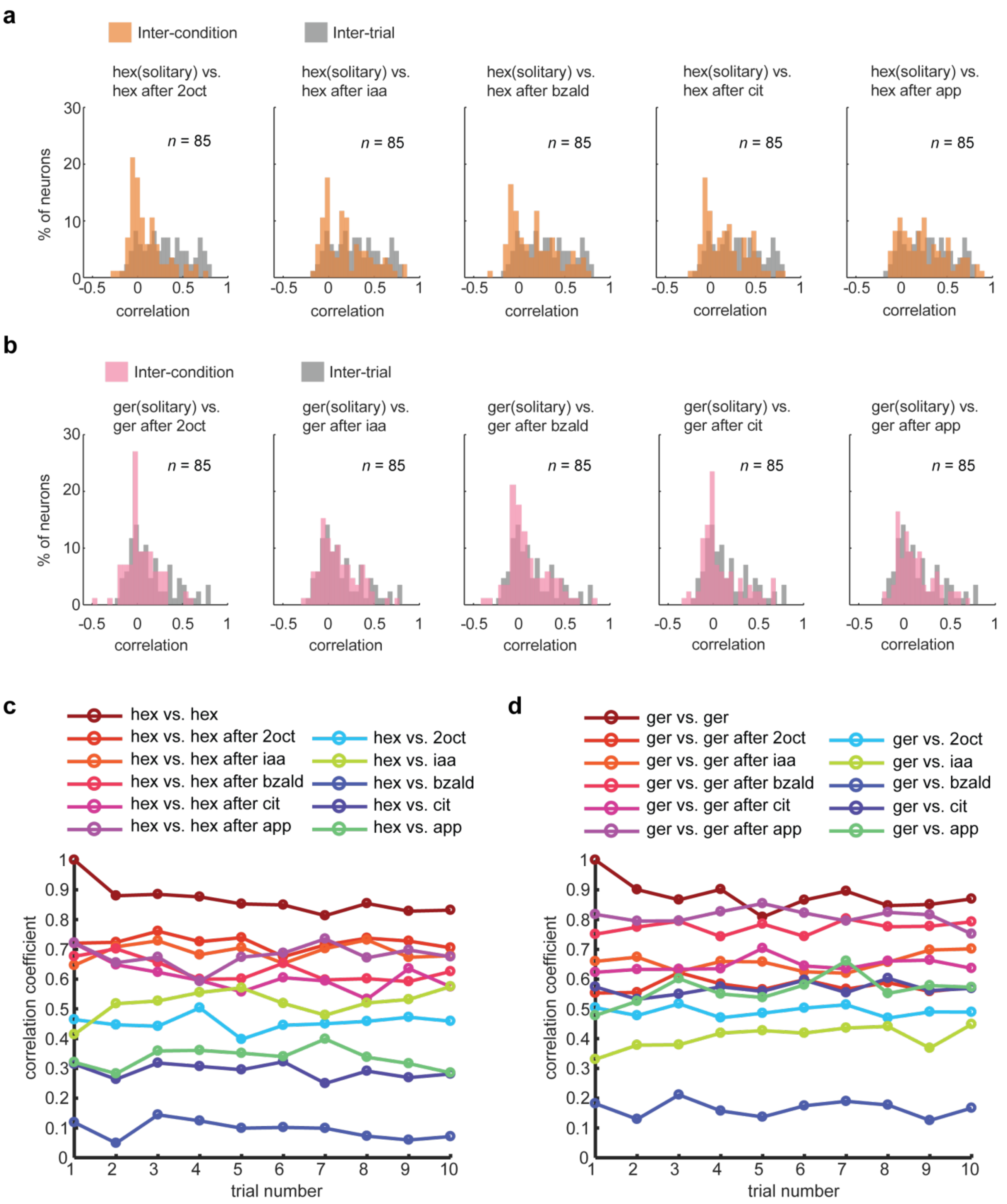

Supplementary Figure 12

(a) Distributions of correlation values between inter-condition (colored) and inter-trial (gray) PN spike trains are shown. This analysis is similar to the one done for generating **Fig. 4e, f**. For computing correlation between trials, for each PN, we compared the similarity between the mean PN responses in the first five trials with the mean response in the remaining five trials of

hex(solitary) stimulus. To allow for a fair comparison, the correlations for inter-condition correlations (plotted in orange) were calculated by estimating the similarity between the mean PN response in the first five trials of hex(solitary) exposures with the mean response in the first five trials of sequential hex presentations. This was again done for each PN and for each sequential hex presentation to generate the five orange distributions shown in the plot.

(b) Similar plots as in **panel a** but analyzing PN responses to geraniol.

(c) Comparison of combinatorial PN response profiles activated by the same odorant across trials, same odorant across stimulus histories, and between different odorants is shown as a function of trial number. Note that all comparisons are made with respect to the ensemble PN responses elicited by solitary presentation of hexanol in the very first trial.

(d) Similar plots as **panel c** but plotted when the target odor is ger.

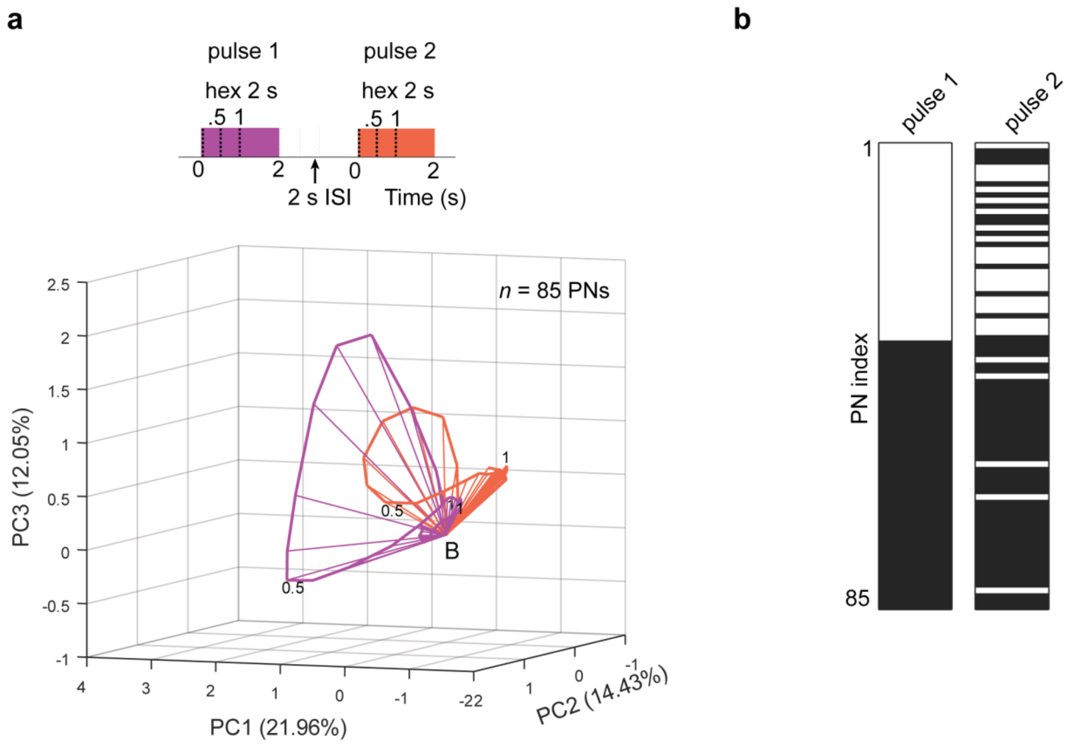

Supplementary Figure 13

(a) Similar trajectory plot as shown in **Fig. 2a** but comparing the population PN responses generated during the presentation of two hexanol pulses. The second pulse of hexanol was presented 2 s after the termination of the first pulse. These data were re-analyzed from our previous study[2].

(b) Similar barcodes as in **Fig. 3a** but identifying the responsive PNs in the first and the second pulse.

REFERENCES:

1 Saha, D. *et al.* A spatiotemporal coding mechanism for backgroundinvariant odor recognition. *Nature neuroscience* **16**, 1830-1839, doi:10.1038/nn.3570 (2013).

2 Saha, D. *et al.* Engaging and disengaging recurrent inhibition coincides with sensing and unsensing of a sensory stimulus. *Nature communications* **8**, 15413, doi:10.1038/ncomms15413 (2017).
